# Light-Enhanced Cytotoxicity of Doxorubicin by Photoactivation

**DOI:** 10.3390/cells12030392

**Published:** 2023-01-21

**Authors:** Giulia Greco, Luca Ulfo, Eleonora Turrini, Alessia Marconi, Paolo Emidio Costantini, Tainah Dorina Marforio, Edoardo Jun Mattioli, Matteo Di Giosia, Alberto Danielli, Carmela Fimognari, Matteo Calvaresi

**Affiliations:** 1 Dipartimento di Chimica “Giacomo Ciamician”, Alma Mater Studiorum—Università di Bologna,40126 Bologna, Italy; 2 Dipartimento di Farmacia e Biotecnologie, Alma Mater Studiorum—Università di Bologna, 40126 Bologna, Italy; 3 Dipartimento di Scienze per la Qualità della Vita, Alma Mater Studiorum—Università di Bologna, 47921 Rimini, Italy

**Keywords:** doxorubicin, photochemotherapy, photosensitizer, photodynamic therapy, photoactivation, reactive oxygen species

## Abstract

The combination of photodynamic therapy with chemotherapy (photochemotherapy, PCT) can lead to additive or synergistic antitumor effects. Usually, two different molecules, a photosensitizer (PS) and a chemotherapeutic drug are used in PCT. Doxorubicin is one of the most successful chemotherapy drugs. Despite its high efficacy, two factors limit its clinical use: severe side effects and the development of chemoresistance. Doxorubicin is a chromophore, able to absorb light in the visible range, making it a potential PS. Here, we exploited the intrinsic photosensitizing properties of doxorubicin to enhance its anticancer activity in leukemia, breast, and epidermoid carcinoma cells, upon irradiation. Light can selectively trigger the local generation of reactive oxygen species (ROS), following photophysical pathways. Doxorubicin showed a concentration-dependent ability to generate peroxides and singlet oxygen upon irradiation. The underlying mechanisms leading to the increase in its cytotoxic activity were intracellular ROS generation and the induction of necrotic cell death. The nuclear localization of doxorubicin represents an added value for its use as a PS. The use of doxorubicin in PCT, simultaneously acting as a chemotherapeutic agent and a PS, may allow (i) an increase in the anticancer effects of the drug, and (ii) a decrease in its dose, and thus, its dose-related adverse effects.

## 1. Introduction

Photodynamic therapy (PDT) is a minimally invasive therapeutic modality currently approved for the treatment of different types of cancers [1,2]. PDT is based on three main elements: a compound with photosensitizing properties (photosensitizer, PS), light, and oxygen. Briefly, light irradiation excites the PS, which in turn generates locally reactive oxygen species (ROS) responsible for its cytotoxic effects [1,2]. Two types of photochemical pathways can be triggered, type I and type II. In the type I mechanism, the excited PS directly interacts with biomolecules and produces radicals that eventually react with oxygen, generating cytotoxic ROS. In type II reactions, instead, the excited PS forms singlet oxygen (^1^O_2_) by directly transferring its energy to ground-state oxygen ^3^O_2_. The generated ROS interact with various biomolecules, such as DNA, proteins, and membranes, causing oxidative damage and leading to cell death [1,2]. PDT can also exert anticancer effects by damaging the tumor vasculature or stimulating antitumor immune responses, increasing its beneficial therapeutic potential [1,2].

**Scheme 1:**
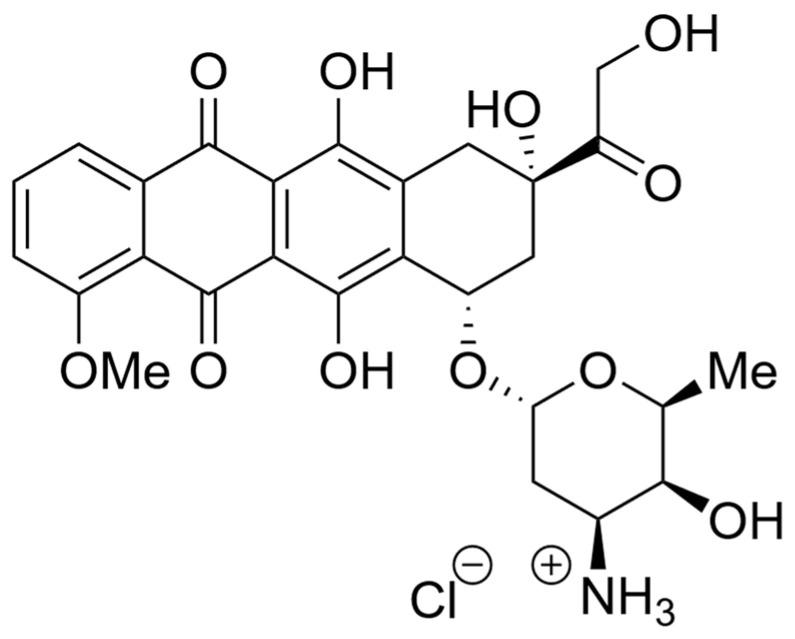
Chemical structure of doxorubicin.

PDT treatment can be combined with chemotherapy (photochemotherapy, PCT) leading to additive or synergistic actions, which are observed both in vitro and in vivo [3]. Furthermore, PCT proved effective against tumor cells resistant to anticancer drugs [3,4]. Doxorubicin belongs to the anthracycline family (Scheme 1) and it is one of the most potent anticancer drugs, widely used to treat hematologic and solid cancers [5,6,7]. The anticancer activity of doxorubicin relies upon three main mechanisms: intercalation of DNA, inhibition of topoisomerase II, and the generation of ROS [5,6,7]. The cytotoxic activity of doxorubicin ultimately leads to DNA, membrane, and protein damage, which altogether results in cancer cell death.

Despite its high efficacy, two factors limit the clinical use of doxorubicin: severe side effects such as cardiotoxicity and the development of chemoresistance [5,6,7].

Previous studies demonstrated that PCT based on the combination of doxorubicin with different PSs exhibits higher anticancer effects than with single agents [8,9,10,11,12,13,14,15,16], enhancing drug delivery to tumors [17] and overcoming tumor drug resistance [18].

In addition, the synergistic action of PCT allows a decrease in the required dose of doxorubicin and thus of its dose-related adverse effects, including anthracycline cardiotoxicity [11,13].

However, the mechanisms responsible for the synergistic PCT anticancer effects have not been fully elucidated [12]. Moreover, it is not clear if the best anticancer effects are obtained after concomitant administration of doxorubicin and PS [19], or when doxorubicin is administered after PDT [11].

Since PCT is based on the administration of two different compounds, many problems can arise due to the different pharmacokinetic and/or pharmacodynamic profiles of the two molecules.

Due to its extensively conjugated molecular structure, doxorubicin is a chromophore able to absorb light in a wide range of the visible spectrum [20]. This characteristic makes doxorubicin a potential PS [21,22,23,24]. Differently from previous studies that investigated the potential of doxorubicin-based PCT in combination with a second PS, here, we exploited the intrinsic photosensitizing properties of doxorubicin [21,22,23,24] to explore whether the chemotherapeutic drug per se could serve also as a PS in PDT. Following photoactivation, a light-dependent generation of ROS and an increase in the doxorubicin cytotoxic activity were observed.

## 2. Materials and Methods

### 2.1. Computational Details

The computations were carried out using Gaussian16 [25]. Doxorubicin tautomers were optimized using density functional theory (DFT), the B3LYP hybrid functional, and the 6-311+G** basis set (B3LYP/6-311+G**) [26,27]. Frequency calculations were carried out on the optimized geometries to check the nature of the critical points. The structure of the most stable tautomer was used to calculate the Jablonski diagram of doxorubicin. TD-DFT calculations [28] were carried out to determine the S_1_ state energy, the molecular orbitals involved in the electronic transitions, and the UV–Vis spectrum of the doxorubicin. All reported calculations were carried out using water as a solvent. The solvent effect was taken into account using the IEF-PCM solvation model [29].

### 2.2. Quantification of Reactive Oxygen Species

The ABMDMA assay consists of a colorimetric test for the selective detection of ^1^O_2_ [30,31,32]. The molecular probe, 9,10-anthracenediyl-bis (methylene) dimalonic acid (ABMDMA), shows a characteristic absorption band in the UV range (from 320 to 420 nm). The selective reaction with ^1^O_2_ produces the corresponding endoperoxide, a molecular species that does not absorb in the same spectral range [31,32,33]. Monitoring the reaction by UV–Vis spectrophotometry allows the quantification of ^1^O_2_ produced by the PS after irradiation [30,31,32].

A stock solution of doxorubicin 2 mM was prepared in phosphate buffer saline (PBS) with 5% DMSO. Different concentrations of doxorubicin (5, 10, 20, and 40 µM) were prepared from stock using PBS dissolved in D_2_O. Next, 97 µL of each sample were loaded into wells (96-multiwell plate), and 3 µL of 5 mM ABMDMA stock solution dissolved in DMSO were added to each well. The multiwell plate was exposed to the light source (cold white LED, Valex), at a distance of 19 cm from the cell plate surface (irradiance = 24 mW/cm^2^, measured with a photo-radiometer Delta Ohm LP 471 RAD) [33].

The absorbance of the samples was recorded at 380 nm before and after irradiation using the EnSpire^®^ Multimode Plate Reader (PerkinElmer, Waltham, MA, USA). The estimation of ^1^O_2_ produced is proportional to the decrease in the initial absorbance of ABMDMA (150 µM).

The Amplex Red assay is based on the reaction between the colorless Amplex Red (N-acetyl-3,7-dihydroxyphenoxazine) probe with peroxides forming a chromophoric species, the resorufin [30,34,35,36]. This enzymatic reaction is catalyzed by horseradish peroxidase (HRP). The doxorubicin solutions were prepared in 50 mM phosphate buffer at pH 7.4 (PB) at the same concentrations used for the ABMDMA test. A working solution (WS) was prepared by adding 10 µL of the stock solution of Amplex Red (50 mM in DMSO) into 1 mL of PB to obtain a final concentration of 500 µM and then adding 10 µL of 0.4 mg/mL of HRP dissolved in PB. Next, 90 µL of the samples were loaded into the wells (96-multiwell plate); the plate was irradiated under the same conditions used for the previous assay. A quantity of 10 µL of WS was added to each sample, activating the reaction between Amplex Red and the hydrogen peroxide (H_2_O_2_) produced upon irradiation. The plate was kept in incubation for 30 min, in dark conditions at room temperature.

The absorbance of the samples was measured at 560 nm before the addition and after the incubation with Amplex Red. The same analysis was performed in a corresponding plate kept in dark. All of the measurements were performed using an EnSpire^®^ Multimode Plate Reader (PerkinElmer).

To convert the absorbance values to the H_2_O_2_ concentration, a calibration curve produced using standard solutions of H_2_O_2_ was used.

The contribution of the H_2_O_2_ produced by the samples kept in dark was subtracted from the H_2_O_2_ concentration estimated for the corresponding irradiated samples.

### 2.3. Cell Culture

Human acute T leukemia (Jurkat), human epidermoid carcinoma (A-431), estrogen (ER) and progesterone (PR) receptor-positive human breast cancer (MCF-7), and ER, PR, and epidermal growth factor receptor-2 (HER2)-negative human breast cancer (MDA-MB-231) cells were obtained from LGC Standard (LGC Group, Middlesex, UK).

Jurkat, A-431, and MDA-MB-231 cells were cultured in Roswell Park Memorial Institute (RPMI) 1640 medium, supplemented with 10% heat-inactivated fetal bovine serum (FBS), 1% L-glutamine 200 mM, and 1% penicillin (10,000 units)/streptomycin (10 mg/mL) solution (all provided by Euroclone, Pero, Italy). MCF-7 cells were propagated in Dulbecco’s modified Eagle’s medium (DMEM, Euroclone) supplemented with 10% FBS, 1% L-glutamine 200 mM, 1% penicillin/streptomycin, and 0.1% insulin. All cells were maintained at 37 °C under 5% CO_2_ in a humidified incubator.

### 2.4. Cell Treatment and Irradiation

Exponentially growing cells were treated with increasing concentrations of doxorubicin (1.25–40 µM) for 2.5 h. At the end of incubation, cells were washed twice with PBS 1X and irradiated in PBS 1X with a low-irradiance white light LED (24 mW/cm^2^) for 30 min. To assess the contribution of photoirradiation in the cytotoxic effects of doxorubicin, cells were exposed to the same concentration of chemotherapeutic drug but kept in the dark. Following irradiation or dark incubation, PBS was removed, and cells were cultured in drug-free complete medium for 24 h.

### 2.5. Cell Viability Assays

To assess cell viability, 100,000 Jurkat cells were treated as previously described. After irradiation or dark incubation, Jurkat cells were analyzed using SYTOX™ Green Nucleic Acid Stain (Thermo Fisher Scientific, Waltham, MA, USA). Briefly, an aliquot of cells was diluted in PBS 1X containing 10 nM of the fluorescent dye, incubated for 20 min at room temperature in the dark, and then analyzed by flow cytometry using a Guava EasyCyte 6 2L cytometer (Guava Technologies, Merck Millipore, Darmstadt, Germany). At least 10,000 events were recorded for each sample. The percentage of viable cells was calculated by normalizing the fluorescence of treated samples on the untreated cells.

For adherent cells MCF-7, MDA-MB-231, or A-431, 10,000 cells were seeded in triplicate for each experimental condition. After light irradiation for 30 min or dark incubation and recovery in drug-free complete medium for 24 h, cell viability was assessed spectrophotometrically using 4-methylumbelliferyl heptanoate assay (MUH; Sigma Aldrich, Merck, St. Luis, MO, USA). MUH becomes highly fluorescent after hydrolysis of the ester linkage and measures cellular lipase and esterase activity that is proportional to cell viability. After treatment, cells were washed with PBS 1X and then incubated with MUH 0.01 mg/mL for 30 min at 37 °C and 5% CO_2_. Fluorescence (330 nm excitation; 450 nm emission) was measured using a Victor X3 microplate reader (Perkin Elmer). The percentage of living cells was calculated by normalizing the fluorescence of treated samples to the untreated cells.

### 2.6. Microscopic Analysis

To assess doxorubicin uptake and intracellular localization, Jurkat or MDA-MB-231 cells were incubated with doxorubicin 20 µM for 0, 0.5, 1, 1.5, 2, and 2.5 h. Briefly, 30,000 MDA-MB-231 cells were seeded on round coverslips and after overnight incubation were treated as previously described. After treatment, 70,000 Jurkat cells were prepared for microscopic analysis using a Cytospin4 (Thermo Shandon, Cambridge, UK), centrifuging at 2000 rpm for 5 min on the microscope slide. Before the analysis, Hoechst 33342 (Sigma Aldrich) was added to the samples at a final concentration of 1 µg/µL. Cells were analyzed using a Nikon A1R confocal microscope (Nikon, Tokyo, Japan).

Images were elaborated using Fiji software and nuclear colocalization was detected using a “colocalization finder” plug-in that measures the pixel fluorescence on each channel and overlaps the signal, automatically calculating the Pearson’s coefficient, indicated with r, which considers the gray value of pixels of each channel and the average intensity over the full image [37]. A value of r = 1 indicates complete colocalization.

### 2.7. Analysis of Caspase-3 Activity

The analysis of caspase-3 activity was performed using a caspase-3 colorimetric assay kit (Enzo Life Sciences, New York, NY, USA), according to the manufacturer’s instructions. This kit exploits a caspase 3-specific substrate, the amino acid sequence Ac-Asp-Glu-Val-Asp (Ac-DEVD), conjugated to the chromophore p-nitroaniline (pNA). In the presence of active caspase-3, Ac-DEVD is cleaved from the chromophore and the absorbance of the free pNA is quantified spectrophotometrically. Briefly, 3 × 10^6^ Jurkat and 1 × 10^6^ MDA-MB-231 cells were treated with 20 µM doxorubicin for 2.5 h and then irradiated for 30 min. To assess caspase activation independently from photoactivation of the drug after 24 h of doxorubicin treatment, cells were treated according to the same condition but left in the dark. After 24 h incubation in drug-free complete medium, both irradiated and nonirradiated cells were lysed using cell lysis buffer included in the kit, and the protein concentration of the cellular lysates was quantified with a Bradford assay. Up to 150 µg of proteins were incubated for 2 h at 37 °C in the dark in a 2X reaction buffer containing DTT (dithiothreitol) 10 µM and Ac-DEVD-pNA substrate 200 µM. Absorbance was measured at 405 nm using a Victor X3 microplate reader. Caspase-3 activity was calculated as the fold increase in treated cells compared to untreated cells.

### 2.8. Necrotic Cell Death Determined by SYTOX™ Green Nucleic Acid Stain

Necrotic events were determined by SYTOX™ Green staining. Jurkat and MDA-MB-231 cells were seeded and treated with doxorubicin 20 μM, as previously described. Cells underwent light irradiation for 30 min or dark incubation and recovery in drug-free complete medium for 24 h. MDA-MB-231 cells were trypsinized using TrypLE™ Express Enzyme (1X) (Thermo Fisher Scientific). Jurkat and MDA-MB-231 cells were suspended in PBS 1X containing SYTOX™ Green 10 nM. After incubation for 20 min at room temperature in the dark, the cells were analyzed by flow cytometry using a Guava EasyCyte 6 2L cytometer (Guava Technologies). The percentage of SYTOX™ Green-positive cells was calculated by normalizing the fluorescence of treated samples to the untreated cells.

### 2.9. Intracellular ROS Generation

The luminescent assay ROS-Glo™ (Promega, Madison, WI, USA) was used to assess intracellular ROS generation. The kit provides a derivatized luciferin substrate that reacts directly with H_2_O_2_ to generate a luciferin precursor. The subsequent addition of ROS-Glo™ Detection Solution converts the precursor to luciferin and provides Ultra-Glo™ Recombinant Luciferase that produces a light signal proportional to the level of H_2_O_2_ present in the sample. Briefly, 30,000 Jurkat or MDA-MB-231 cells were treated in complete medium with 20 or 40 µM doxorubicin for 2.5 h. After incubation, cells were washed twice with PBS 1X to remove the excess drug and then irradiated for 30 min in PBS 1X. In parallel, to measure ROS generation by doxorubicin in dark conditions, cells were treated according to the abovementioned conditions and kept in dark. After irradiation or dark incubation, 20 µL of substrate solution was added and incubated with the cells for 20 min at 37 °C, then 100 µL of detection solution was added to the cells and incubated at room temperature for 20 min. Luminescence was recorded using an EnSpire^®^ Multimode plate reader (PerkinElmer, Waltham, MA, USA). Results are expressed as a fold increase compared to untreated cells.

### 2.10. Statistical Analysis

Results are expressed as the mean ± SEM of at least three independent experiments, unless differently specified. Statistical analyses were performed using paired *t*-test or one- or two-way ANOVA within Tukey or Dunnet as post-tests. IC_50_ values (concentrations that inhibit 50% of cell viability) were calculated from nonlinear regression curves. The statistical software GraphPad InStat 8.0 version (GraphPad Prism, San Diego, CA, USA) was used, and *p* < 0.05 was considered significant.

## 3. Results and Discussion

### 3.1. Light-Dependent Generation of ROS by Doxorubicin

**Figure 1:**
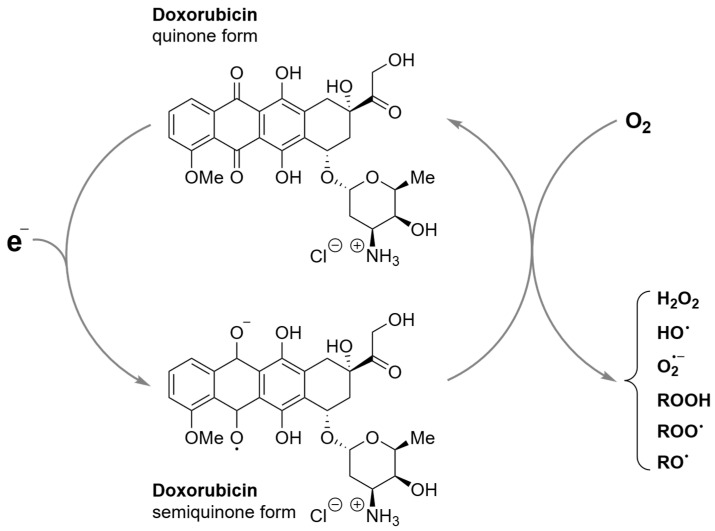
Redox cycling of doxorubicin. One-electron reduction of the quinone moiety mediated by the cellular oxidoreductases results in the formation of a semiquinone radical that regenerates the parent quinone by reducing an oxygen molecule to a superoxide radical. This initiates a reaction cascade leading to the formation of other ROS.

It is well known that doxorubicin can enzymatically generate ROS (Figure 1) [7]. Several oxidoreductases, such as cytochrome P450 reductases, xanthine oxidase, and NADH dehydrogenase (complex I) of the mitochondrial electron transport chain, can convert the quinone moiety of doxorubicin into a semiquinone [38]. This semiquinone then swiftly regenerates, converting O_2_ into ROS, such as superoxide anion and H_2_O_2_, that can be subsequently converted, generally via the Fenton reaction, into hydroxyl radicals [39].

This mechanism, which is implicated in the anticancer activity of the drug, is specific and has been suggested to account for the cardiotoxicity of doxorubicin [40].

**Figure 2:**
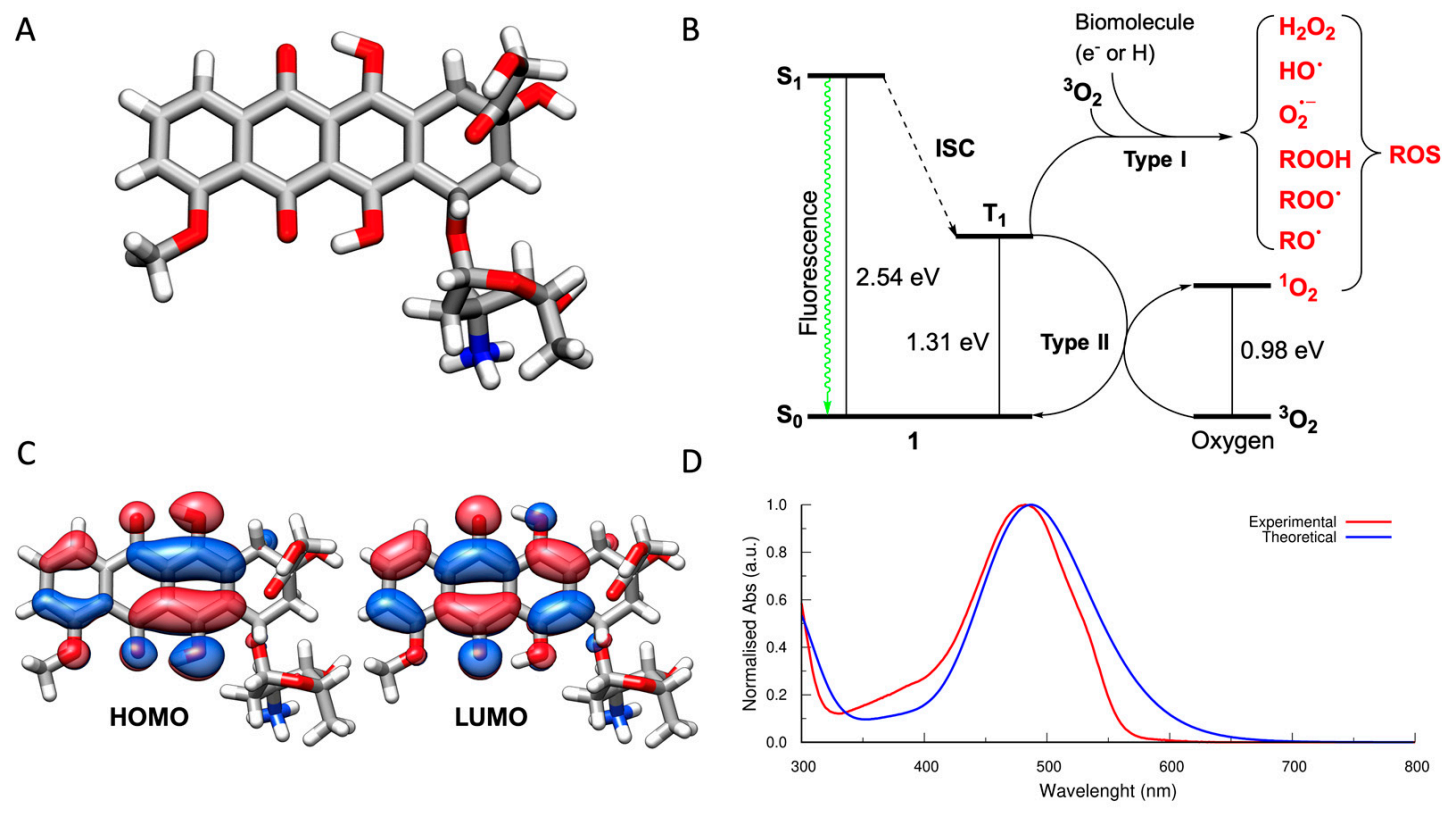
(**A**) A 3D representation of the most stable tautomer of the doxorubicin molecule. (**B**) Jablonski diagram of doxorubicin. (**C**) HOMO and LUMO of doxorubicin. (**D**) Experimental (red line) and calculated UV–Vis spectra of doxorubicin.

Here we propose a controllable modality to produce ROS by doxorubicin, inducing the generation of ROS upon irradiation. Upon focused irradiation, light can selectively trigger the local generation of ROS, following photophysical pathways. To check the feasibility of our approach, we investigated the excited states of doxorubicin, carrying out time-dependent density functional theory (TD-DFT) calculations. The most stable tautomer of doxorubicin was identified (Figure 2A) and its excited states were calculated (Figure 2B).

When irradiated with a white light, doxorubicin can absorb a photon and be promoted from its ground state (S_0_) to the first singlet excited state (S_1_). The S_0_–S_1_ excitation transition is a π–π^∗^ excitation, totally ascribed to the HOMO–LUMO orbitals that are delocalized over the anthracene framework of doxorubicin (Figure 2C). S_1_ lays 2.54 eV (487.7 nm) above S_0_, in excellent agreement with UV–Vis data (482.0 nm). In Figure 2D the experimental and the calculated UV–Vis spectra are reported. Calculations reproduce with great accuracy the experimental UV–Vis spectrum, vouching for the accuracy of the computations. Doxorubicin from the S_1_ excited state can decay to the ground state S_0_ through a radiative process, generating the typical fluorescence of the doxorubicin molecule (Figure 2B). An alternative pathway may occur from S_1_ to T_1_ via intersystem crossing (ISC), populating the T_1_ state (Figure 2B). Due to the spin-forbidden deactivation process to the ground state, the T_1_ excited state is generally characterized by a longer lifetime than S_1_, allowing an efficient interaction with molecular oxygen (^3^O_2_), generating ROS through two different pathways. The type I mechanism is characterized by an electron-transfer process, while the type II mechanism is characterized by an energy-transfer process.

The Jablonski diagram of doxorubicin (Figure 2B) suggests that (i) it may convert from S_1_ to T_1_ via ISC, opening the path to ROS generation, and (ii) T_1_ may generate ^1^O_2_ because it is energetically located above the ^1^O_2_ state.

**Figure 3:**
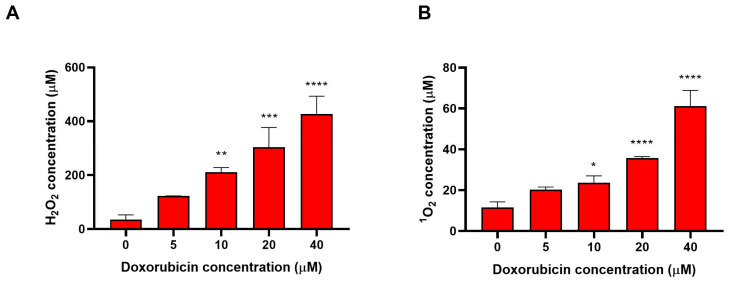
Quantification of ROS generated through type I (**A**) and type II (**B**) mechanisms as a function of doxorubicin concentration after 30 min of white light irradiation. (**A**) Amplex Red assay for H_2_O_2_ detection. (**B**) ABMDMA assay for ^1^O_2_ detection. Results are expressed as mean ± SD. * *p* < 0.05; ** *p* < 0.01; *** *p* < 0.001; **** *p* < 0.0001 vs. control (doxorubicin 0 μM).

To confirm the photosensitizing potential of doxorubicin, the ABMDMA and the Amplex Red assays were used. Doxorubicin showed a concentration-dependent ability to generate ROS and ^1^O_2_ (Figure 3) upon irradiation. This means that doxorubicin can be used as a PS.

In principle, this light-dependent ROS-generation activity provides the opportunity to enhance the cytotoxic action of doxorubicin, with a focused irradiation (localized) at the desired site of action, lowering the side effects of the drug on nonirradiated off-target tissues [41]. To overcome the unfavorable absorption of doxorubicin in only the 450–500 nm region, characterized by very low penetration of the tumor tissues, (i) the conjugation of doxorubicin with antenna systems, such as NIR-excited upconverting nanoparticles (UCNPs) [42] can be utilized to activate ROS generation in considerably deeper tissues because of the strong tissue penetration capabilities of NIR light, and (ii) interventional techniques with optical fibers and endoscopy can irradiate doxorubicin directly inside the tumor tissue, i.e., interstitial PDT (I-PDT) [43].

### 3.2. Photoactivation Enhances the Cytotoxic Activity of Doxorubicin

To understand the anticancer potential of doxorubicin-based PCT, we tested its cytotoxic activity and phototoxicity in a panel of cancer cell lines representing both hematologic and solid tumors, i.e., Jurkat, A-431, MCF-7, and MDA-MB-231. Incubation for 2.5 h with doxorubicin caused a significant decrease in cell viability on all tested cell lines kept in the dark, due to the well-known chemotherapeutic action.

**Figure 4:**
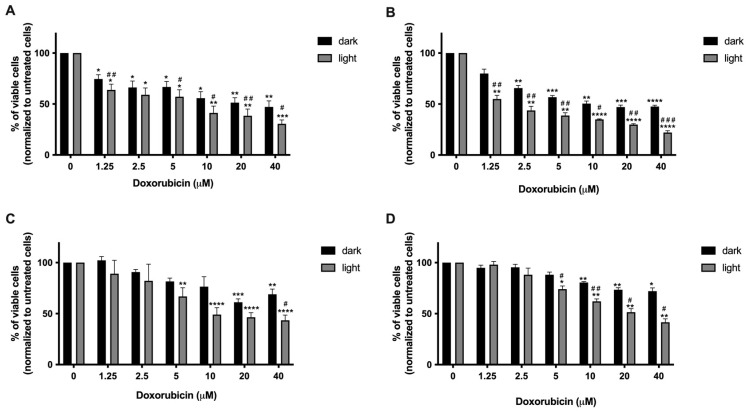
Percentage (%) of Jurkat (**A**), A-431 (**B**), MCF-7 (**C**), and MDA-MB-231 (**D**) viable cells after treatment with doxorubicin for 2.5 h followed or not by 30 min light irradiation and 24 h recovery in drug-free complete medium. * *p* < 0.05; ** *p* < 0.01; *** *p* < 0.001; **** *p* < 0.0001 vs. untreated cells. # *p* < 0.05; ## *p* < 0.01; ### *p* < 0.001 vs. nonirradiated cells.

The photoactivation of doxorubicin significantly decreased cell viability starting from the lowest tested concentrations (Figure 4). At the highest dose of the chemotherapeutic drug (40 µM), we observed the most significant differences. In MDA-MB-231 cells, the PDT treatment determined a further 30% reduction in cell viability. 

The different sensitivity of cells treated with doxorubicin and exposed to light irradiation vs. cells kept in the dark was quantitatively evaluated based on IC_50_ values (Table 1). The photoactivation of doxorubicin led to IC_50_ values 4.2-, 9.9-, 4.7-, and 7.3-fold lower than those obtained in dark incubation in Jurkat, A-431, MCF-7, and MDA-MB-231 cells.

To elucidate the mechanisms triggering the cytotoxic effects observed in our experimental setting, we carried out additional experiments on Jurkat and MDA-MB-231 cells, because of (i) the clinical use of doxorubicin for leukemias and breast carcinomas [44] and (ii) the evidence that doxorubicin is one of the few treatment options for triple-negative breast carcinoma, which represents one of the most aggressive breast cancers and is associated with worst prognosis compared to the hormone-positive tumors [45,46].

### 3.3. Uptake and Nuclear Localization of Doxorubicin

ROS generated by PDT treatments have a limited life span in cells, so the photodamage is generally restricted to the cellular regions where the PS is localized and generates ROS [47]. Therefore, it is evident that PDT efficiency is significantly influenced by the subcellular localization of the PS [47].

As a consequence, many investigations dealt with the use of organelle-targeted PSs to enhance the effect of PDT [47]. The most important organelles targeted by PDT are cell membranes, lysosomes, mitochondria, endoplasmic reticulum, and nucleus [47]. These organelles are essential for preserving cell morphology and function and their damage induces cellular malfunction, apoptosis, or necrosis.

Targeting the nucleus to harm the cancer cell is a very effective strategy in PDT. Targeting chlorin e6, one of the most used PS, to the nucleus increases its photosensitizing activity by a factor of over 2000-fold, demonstrating the high sensitivity of the nucleus to photodynamic activity [48]. Typically, PSs are targeted to the nucleus via bioconjugation with peptides or aptamers [47].

We examined the doxorubicin intracellular uptake and localization in MDA-MB-231 and Jurkat cells.

**Figure 5:**
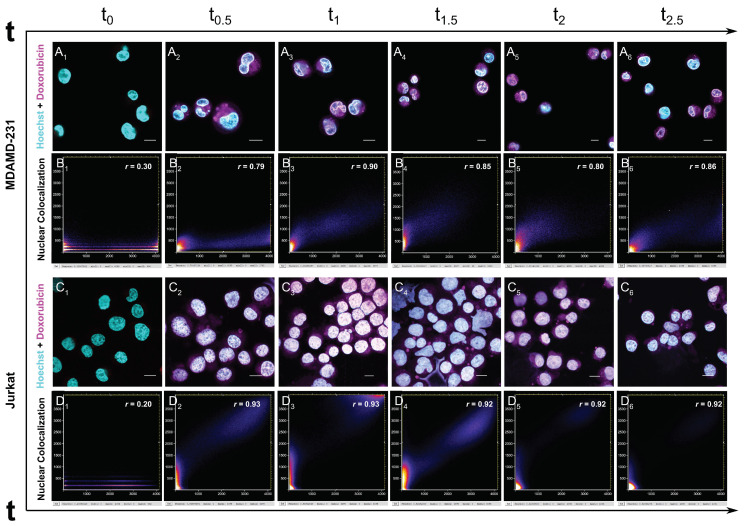
Cellular uptake and nuclear colocalization of doxorubicin in MDA-MB-231 and Jurkat cells treated with doxorubicin (20 μM) for 0, 0.5, 1, 1.5, 2, and 2.5 h. (**A**,**C**) confocal microscopy images of internalized doxorubicin (magenta) and nuclei (cyan) at different time points. (**B**,**D**) scatter plot of doxorubicin nuclear colocalization calculated by the colocalization finder plug-in for ImageJ, r is Pearson’s correlation coefficient and r = 1 indicates complete colocalization.

In both MDA-MB-231 and Jurkat cells, doxorubicin was internalized and immediately started to localize in the cells’ nuclei after 30 min of incubation (Figure 5), with a high degree of nuclear colocalization in both cell lines.

Considering that doxorubicin localizes rapidly in the nucleus [49,50] without the need for any external targeting tag, its nuclear localization per se is an added value for its use as a photoactivated anticancer drug.

### 3.4. Photoactivated Doxorubicin Induces Both Caspase-Dependent and -Independent Cell Death

**Figure 6:**
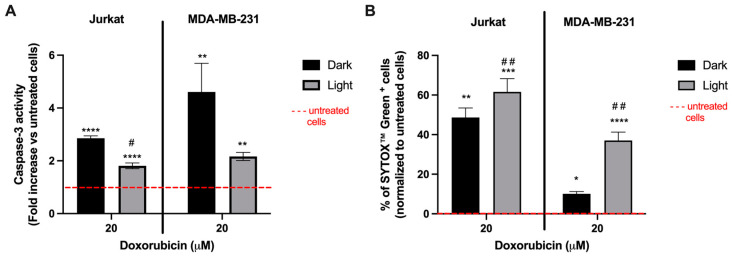
Caspase-3 activity (**A**) and percentage (%) of SYTOX™ Green-positive cells (**B**) in Jurkat and MDA-MB-231 cells after treatment with doxorubicin (20 μM) for 2.5 h followed or not by light irradiation for 30 min and 24 h recovery in drug-free complete medium. * *p* < 0.05; ** *p* < 0.01; *** *p* < 0.001; **** *p* < 0.0001 vs. untreated cells. # *p* < 0.05; ## *p* < 0.01 vs. nonirradiated cells.

The ability of doxorubicin to induce apoptotic cell death in tumor cells is well-established [6,51,52,53]. Caspase-3 is one of the main biomarkers used to evaluate apoptosis induction [54]. Therefore, to assess whether the enhanced cytotoxicity of doxorubicin induced by light irradiation was proportional to an increased triggering of the apoptotic cell death pathway, caspase-3 activity was analyzed. As shown in Figure 6A, caspase-3 activity increased 2.85-fold in nonirradiated Jurkat cells after exposure to doxorubicin for 2.5 h, while a lower caspase-3 activity (1.81-fold) was observed in light irradiated cells (Figure 6A). A similar trend was recorded in MDA-MB-231 cells (Figure 6A).

These results suggest that photoactivated doxorubicin may trigger other cell death pathways, different from caspase-dependent apoptosis. Along with a decreased caspase-3 activity, after light irradiation, we recorded an increase from 46.68% to 61.58% and from 10.09% to 37.05% in Jurkat and MDA-MB-231 cells, respectively, in the SYTOX™ Green-positive population (Figure 6B). SYTOX™ Green is a fluorescent dye binding nucleic acid that is impermeant to living cells but penetrates compromised membranes. It can be used to detect necrotic and late apoptotic cells [55].

These results indicate an increase in the loss of membrane integrity in irradiated cells, a key morphological feature of cells undergoing necrotic cell death [56]. Thus, the decrease in caspase-3 activity and the increase in cells with damaged cell membranes recorded after photoactivation suggest that light irradiation potentiates the cytotoxic effects of doxorubicin through the induction of necrotic cell death.

### 3.5. Photoactivated Doxorubicin Generates Intracellular ROS

ROS generation is the main cause of PDT-induced tumor cell death [4]. Therefore, we measured ROS intracellular levels in Jurkat and MDA-MB-231 cells after doxorubicin incubation (2.5 h) followed or not by photoactivation. This measure also determines the effect of a complex matrix on the process of photoactivated ROS generation by doxorubicin.

**Figure 7:**
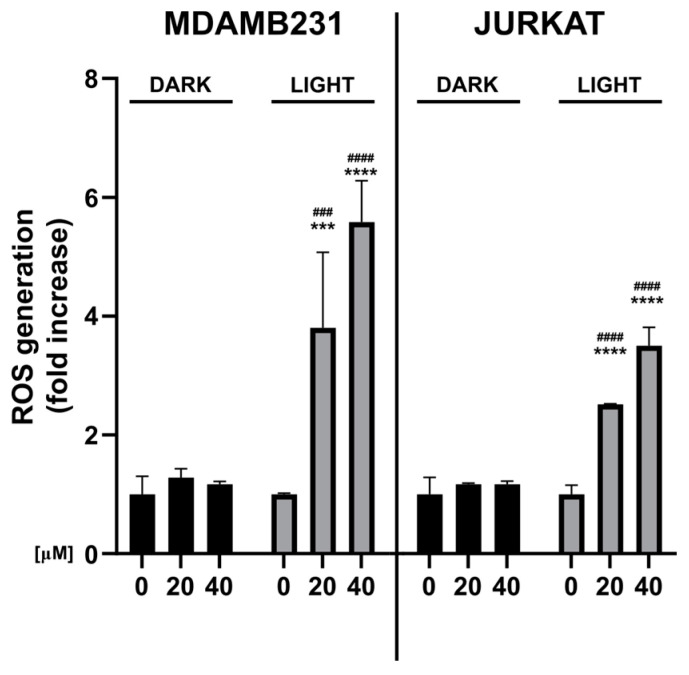
ROS generation in Jurkat and MDA-MB-231 cells after treatment with doxorubicin 20 or 40 µM for 2.5 h followed or not by light irradiation for 30 min. Results are expressed as mean ± SD. *** *p* < 0.001; **** *p* < 0.0001 vs. untreated cells; ### *p* < 0.001; #### *p* < 0.0001 vs. the same concentration of doxorubicin in dark conditions.

Significantly, ROS generation increased only upon PDT treatment (Figure 7); in particular, we recorded a 2.5- and 3.5-fold increase in Jurkat cells treated with 20 or 40 μM doxorubicin, respectively, compared to untreated cells (Figure 7). ROS generation was even higher in MDA-MB-231 cells, with a 3.8- and 5.5-fold increase, respectively (Figure 7). Interestingly, despite the higher generation of ROS, MDA-MB-231 cells were less susceptible to the cytotoxic effects of photoirradiated doxorubicin. This effect could depend on differences in ROS-detoxifying activity between the two cell lines [57,58]. Other mechanisms may be involved in the different sensitivity of tumor cell lines to doxorubicin, including the differential expression of drug efflux pumps that may inversely correlate with drug sensitivity [59].

The levels of intracellular ROS can determine the fate of the cell [60,61]. Indeed, low levels of oxidative stress can induce programmed mechanisms of cell death such as apoptosis, while the accumulation of high levels of ROS can lead to necrotic destruction of the cell [54,61].

## 4. Conclusions

PDT is a modern anticancer strategy able to induce damage and death of the target tumoral cells through multifactorial mechanisms including ROS-mediated damage and killing, and immune system activation. In this paper, we exploited the intrinsic photosensitizer properties of doxorubicin. Altogether, we have shown that photoactivation of doxorubicin generates peroxides and ^1^O_2_ in a light-dependent manner and leads to an increase in its cytotoxic activity. We have also found that doxorubicin readily localizes in the nucleus, without needing external targeting tags, and after photoactivation, triggers different modalities of cell death. The next step of our work will be to explore whether, in addition to the direct cytotoxic effect, doxorubicin-based PDT can induce a form of immunogenic cancer cell death that can be therapeutically exploited to establish protective antitumor immunity.

## Figures and Tables

**Table 1:** IC_50_ values, indicated as mean ± SEM (standard error of the mean), calculated after incubation with doxorubicin for 2.5 h followed or not by 30 min light irradiation and 24 h recovery in drug-free complete medium.

	IC_50_ ± SEM (μM)	
Cell Line	DARK	LIGHT
Jurkat	25.52 ± 9.11	6.12 ± 1.39
A-431	16.75 ± 2.78	1.70 ± 0.27
MCF-7	80.11 ± 32.42	17.05 ± 4.93
MDA-MB-231	161.13 ± 55.12	22.23 ± 2.46

## Data Availability

All data in this study can be requested from the corresponding authors (carmela.fimognari@unibo.it, C.F., and matteo.calvaresi3@unibo.it, M.C.).
